# Prevalence of Laboratory Critical Results in Eye Patients from an Eye Hospital in Southern China

**DOI:** 10.1155/2017/8920350

**Published:** 2017-05-10

**Authors:** Fang Duan, Jingyu Liao, Liping Lin, Xiuping Liu, Kaili Wu

**Affiliations:** Zhongshan Ophthalmic Center, State Key Laboratory of Ophthalmology, Sun Yat-sen University, 54 Xianlie Road, Guangzhou 510060, China

## Abstract

**Objectives:**

To investigate the prevalence of laboratory critical results (CRs) and associated risk factors in patients with eye diseases in a tertiary eye hospital.

**Methods:**

Blood samples were collected from both inpatients and outpatients at Zhongshan Ophthalmic Center, Guangzhou, China, from June 1, 2012, to May 31, 2014, and samples were sent to the hospital's clinical laboratory for blood routine, biochemistry, and blood coagulation tests. Laboratory CRs for blood glucose, sodium, potassium, white blood cell count, platelet count, prothrombin time, fibrinogen, international normalized ratio, and activated partial thromboplastin time were included in the current analysis.

**Results:**

A total of 60403 subjects were enrolled in the current analysis. CRs were identified in 339 tests from 336 patients with a prevalence of 5.7‰. Age was positively associated with the presence of CRs. Compared to patients with lens diseases, patients with strabismus, oculoplastics, and ocular trauma were less likely to have CRs (*P* < 0.05), while patients with tumors were more likely to have CRs (*P* < 0.001).

**Conclusions:**

The prevalence of CRs in eye patients is low but calls for medication attention. It is important for medical personnel, especially ophthalmologists, to increase awareness of the importance, as well as the prevalence and risk factors of CRs.

## 1. Introduction

The concept of critical results (CRs) was first introduced by George D. Lunderberg in 1972 and refers to abnormal laboratory results that suggest a life-threatening situation for the patient if therapy is not promptly initiated [1]. Reporting CRs to the patient care team as rapidly as possible is critical to patient management and prognosis [2]. It has been required by laws and regulations in many countries to report CRs in a timely manner. The Joint Commission on Accreditation of Healthcare Organizations and the College of American Pathologists all contain requirements on CR reporting [3, 4]. The Chinese Hospital Association (CHA) has also established the goal and requirements of reporting CRs [5]. It had been widely accepted that measuring and reporting CRs should be an important part of clinical practice.

Previous studies had assessed CRs in patients with different diseases to guide clinical decision-making [6–8]. However, to the best of our knowledge, the prevalence of CRs in eye patients had never been reported. Eye patients often complain only about their eye problems when they come to the hospital, and it is common for ophthalmologists, as well as other medical staffs, to ignore potential systemic problems of the patients. Failure to identify CRs could harm a patient's prognosis or even lead to significant mortality and morbidity. Thus, it is important to increase awareness of the importance of CRs in specialists to enhance patient care.

Blood tests are needed to obtain CRs. However, the usefulness and necessity of a preoperative blood test, especially for eye patients, are still under debate. In 2000, the New England Journal of Medicine (NEJM) reported that routine blood tests did not significantly decrease complications or improve the outcome for cataract surgery [9]. The American Academy of Ophthalmology guideline also recommended that tests should be ordered only when medical history or physical findings indicate the need [10]. However, China has the greatest population of eye patients and the largest eye surgery quantity; these patients do not have a national medical record and are often lacking awareness of their underlying systemic disease, making it difficult for clinicians to make decisions on whether or not to order blood tests. Is it suitable to order blood tests for all the patients who needed surgery? Thus, the purpose of our study was to evaluate the prevalence of CRs in eye patients and to determine the associated risk factors to help enhance awareness and aid in the clinical decisions of ophthalmologists.

## 2. Methods

### 2.1. Study Population

From June 1, 2012, to May 31, 2014, a total of 60403 inpatients and outpatients were recruited from the Zhongshan Ophthalmic Center (ZOC), Guangzhou. Blood samples were collected and sent for routine blood, biochemistry, and blood coagulation tests at the hospital's clinical laboratory. ZOC is the largest tertiary eye hospital in China, serving over 30,000 inpatients and 1,000,000 outpatients, as well as performing over 35,000 blood sample tests per year.

This study was designed and conducted in accordance with the Helsinki Declaration and was approved by the Institutional Ethics Committee of the Zhongshan Ophthalmic Center, Sun Yat-sen University.

### 2.2. Procedures

All blood samples were collected in a separating gel vacuum tube (BD Diagnostics, USA). Routine blood routine tests were performed with a Sysmex XS-1000i (Sysmex Corporation, Japan), coagulation function tests were performed with a Sysmex CA-7000 (Sysmex Corporation, Japan), and blood biochemical tests were performed by an experienced laboratory technician with a Siemens Dimension RxL Max (Siemens Corporation, German). Following the CHA guidelines [5] and expert recommendations, blood glucose, sodium, potassium, white blood cell count (WBC), platelet count (PLT), prothrombin time (PT), fibrinogen (FIB), international normalized ratio (INR), and activated partial thromboplastin time (APTT) were included in the current analysis as critical results analytes. Following the hospital standard procedures of CR reporting, repeat tests were performed to further verify the results before reporting CRs to the corresponding clinicians. For inpatients, the laboratory technician would inform the patient's bedside clinician, and for outpatients, the laboratory technician would inform either the patient or family members using the contact information in the outpatient electronic medical records system. Details of all phone calls, including time, contact person, and contents, were documented according to agency policy.

All blood test results, as well as information including the patient's name and gender, were recorded in the laboratory information system (LIS) and could be exported into excel files for further analysis. Age was categorized into 9 age groups based on 10-year intervals. Primary ophthalmic diagnoses of the participants were obtained from medical records and further divided into 13 subgroups by an ophthalmologist as follows: cornea disease, lens disorders, glaucoma, uveitis, ocular trauma, strabismus, vitreous/retina disease, optic nerve disease, orbital disease (except for ocular tumor), lacrimal duct disease, oculoplastics disease, ocular tumor, and others.

### 2.3. Statistical Analysis

Chi-square test was used to determine the difference in CR prevalence among categorical variables (age group, gender, and ocular diseases). Univariate logistic regression was used to assess the association between potential risk factors and CRs. Ocular disease subtypes with less than 1000 patients were not included in the regression model. The association between ocular disease subtypes and the prevalence of CRs was assessed after adjusting for age and sex by multiple regression modeling. Statistical analyses were performed using SPSS version 16.0 for Windows. *P* values of ≤0.05 were considered statistically significant.

## 3. Results

**Figure 1:**
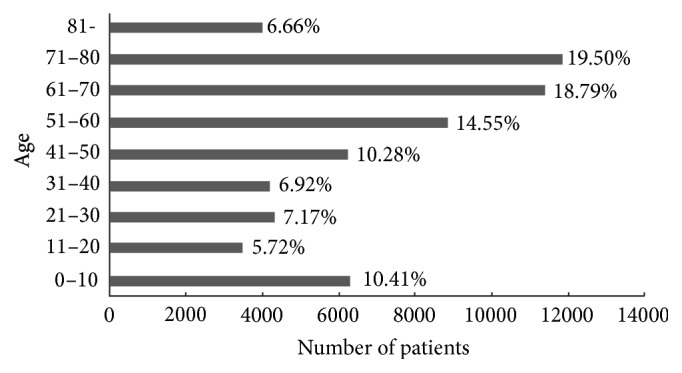
Age-distribution of the study population. In total, 60403 eye patients from a tertiary eye hospital were included.

A total of 60403 eye patients (51.3% male) were included in the current study, with a mean age of 51.2 ± 24.5 years.  Figure 1 shows the age distributions for the population under study. In general, there is a nonnormal distribution for age with nearly 40% of the participants in the 61–80 age group. The patients in the 51–60 age group accounted for approximately 15% of the total patient population. The patients in the younger than 10 age group accounted for approximately 10%, which was similar to the 41–50 age group. The other age groups composed less than 10%. CRs were presented in a total of 339 tests from 336 patients (52.0% male). The prevalence of CRs was 5.7‰.

Table 1 shows the distribution of CRs for the population under study. The most frequently identified CR was abnormal serum potassium, with high potassium recorded in 70 (20.6%) patients and low potassium recorded in 41 (12.1%) patients. The second most common type of CR was PT, which was equal to or more than 20 s in 75 (22.1%) patients. Abnormal blood glucose level was also common, with high glucose in 37 (10.9%) patients and low glucose in 6 (1.8%) patients. High APTT, high PLT, low PLT, low FIB, and high INR were seen in 32 (9.4%), 31 (9.1%), 8 (2.4%), 28 (8.3%), and 5 (1.5%) patients, respectively. High WBC and low sodium were less common, both being identified in only 3 (0.9%) patients.

Table 2 shows the age-, gender-, and disease-specific distributions of CRs. The prevalence of CRs was the highest in the 0–10 age group, followed by the 71–80 and above 80 age groups. The lowest prevalence of CRs was in the 11–20 age group, followed by the 31–40 age group. Women and men had a similar prevalence. As to specific eye diseases, the prevalence of CRs varied from 1.31‰ in patients with strabismus to 19.86‰ in patients with ocular tumors. The prevalence in patients with cornea disease, glaucoma, lens disorder, and retina disease was 8.12‰, 6.51‰, 5.80‰, and 5.25‰, respectively. The prevalence in patients with uveitis, orbital disease, lacrimal duct disease, and strabismus was relatively lower, with values of 1.61‰, 1.41‰, 1.34‰, and 1.31‰, respectively.

Table 3 shows the association between age and eye diseases with CRs. Using subjects aged 71–80 as the reference, patients aged younger than 50 years were more likely to have CRs, except for the 21–30-year-old subgroup. Additionally, using subjects with lens disorders as the reference, patients with strabismus were less likely to have CRs (*P* = 0.002), while patients with ocular tumors were 3.63 times more likely to have CRs (*P* < 0.001).  Table 4 shows the association between individual eye diseases with CRs after adjusting for age and sex. Using patients with lens disorders as the reference, in addition to the two diseases described above, patients with ocular trauma (*P* = 0.003) and oculoplastics disease (*P* = 0.01) were also found to have a lower prevalence of CRs.

## 4. Discussion

To the best of our knowledge, the present study is the first to report on the prevalence of CRs in eye patients and the associated factors. The strength of this study lies in its large sample size and standardized examination methodology. The prevalence of CRs is 5.7‰ in our study, which is lower than that reported in previous studies. Dighe et al. reported the prevalence of CRs as 0.25% at a large academic medical center, and Yang et al. reported the incidence rate of CRs as 0.96% at a large tertiary teaching hospital in China [11, 12]. Previous studies had reported that most CRs were identified at the newborn nursery and adult intensive care unit (ICU), as well as in people with acute kidney injury, cancer, and diarrhea [13, 14]. However, they did not have data focusing on eye patients, which might be due to fewer eye patients and the lower prevalence of CRs of these patients in those general hospitals.

The most commonly identified CR is abnormal potassium, which is inconsistent with the previous studies [11, 13]. As we know, potassium is particularly important for nerve and muscle function and high potassium is related to several health conditions such as type 1 diabetes, Addison's disease, and internal bleeding [15–17]. Critically high potassium levels could lead to paralysis, heart problems, and maybe even death due to heart failure [18, 19]. Patients with symptoms suggesting high potassium should alert ophthalmologists to order blood tests as soon as possible. Thus, it is important for clinicians to identify patients with CRs for high potassium and take prompt action. PT is the second most common CR identified in eye patients. Abnormal PT values indicate bleeding disorders, vitamin K deficiency, or warfarin-containing therapy for the patient and are also a risk factor for surgical complications [20]. Blood tests for PT should also be ordered for patients before operation. Blood glucose, either too low or too high, is another commonly identified CR that could lead to severe clinical outcomes. Diabetes and high blood glucose have been well acknowledged as a risk factor for surgical complications including eye surgeries due to their detrimental effect on blood vessels, nerves, and wound-healing [21–24]. In addition, severe low blood sugar can sometimes be life-threatening, leading to seizures and even coma [25, 26]. Therefore, it is important to recognize CRs for blood sugar and use timely interventions to avoid clinical morbidity and mortality.

There were significant differences in the prevalence of CRs among different age groups. The prevalence of CRs was the highest in the 0–10 age group, suggesting that ophthalmologists should enhance blood testing for this group of patients. Patients at this young age often cannot express themselves well, further adding to the necessity of performing laboratory tests. Older patients also had a higher prevalence of CRs and might also need more attention. In addition, the prevalence of CRs was the highest in patients with ocular tumors (*P* < 0.001). The reason for this might be that tumors are often associated with more severe and systemic diseases, or they may have spread from other parts of the body [27, 28]. This suggests that eye patients with tumors would require collection of a more detailed history of systemic diseases and blood tests. Patients with strabismus (*P* = 0.002), ocular trauma (*P* = 0.003), and oculoplastics disease (*P* = 0.01) were found to have a lower prevalence of CRs. The reasons for this might be that these three diseases are not commonly associated with systemic disease and thus can be deemed as low-risk populations in clinical practice.

Reports from the NEJM and AAO guidelines suggested that preoperative blood tests should only be ordered when medical history or physical examinations indicated the need for them instead of routine preoperative tests for cataract surgery. However, this could not be well applied to developing countries such as China because the majority of patients do not have detailed and uniform medical history records. In addition, the prevalence of CRs in patients with ocular tumor, cornea disease, and glaucoma was higher than in patients with lens disorders, based on our results. In consideration of the importance of adequately recognizing and reporting CRs, we suggest that presurgery CR screening should be instituted in developing countries with special attention to high-risk patients.

Limitations of our study should be noted. As this study is a hospital-based study, the result may not be directly inferable to the general population. Furthermore, only a limited number of eye diseases were included in the analysis. However, we included the most common eye diseases in our studies, which could aid clinical decision-making to a large degree.

## 5. Conclusion

In conclusion, this study demonstrated the prevalence of CRs and its risk factors in eye patients. The prevalence of CRs is 5.7‰ and age was positively associated with the presence of CRs. Compared to patients with lens diseases, patients with strabismus, oculoplastics, and ocular trauma were less likely to have CRs, while patients with tumors were more likely to have CRs.

CRs are by definition very important factors related to an eye patient's treatment and prognosis. The results of our study could offer practical information to help increase the understanding of CRs among ophthalmologists and help them to identify high-risk and low-risk patient populations.

## Figures and Tables

**Table 1:** Distribution of critical results in eye patients by test type.

	Critical results (*n*)	Frequency (%)
Potassium ≥ 6 mmol/l	70	20.6
Potassium ≤ 2.8 mmol/l	41	12.1
Glucose ≤ 2.5 mmol/l	37	10.9
Glucose ≥ 27.8 mmol/l	6	1.8
PT ≥ 20 s	75	22.1
APTT ≥ 48 s	32	9.4
PLT ≤ 30 × 10*E*9/L	31	9.1
PLT ≥ 1000 × 10*E*9/L	8	2.4
FIB ≤ 0.7 g/L	28	8.3
INR ≥ 3.5	5	1.5
WBC ≥ 100 × 10*E*9/L	3	0.9
Sodium ≤ 120 mmol/l	3	0.9
*Total*	339	100

PT: prothrombin time; APTT: activated partial thromboplastin time; PLT: platelet count; FIB: fibrinogen; INR: international normalized ratio; WBC: white blood cell.

**Table 2:** Prevalence of critical results in eye patients by age, gender, and specific eye disease.

	Critical results (−)	Critical results (+)	Prevalence (‰)
Age (years)^**∗**^			
0–10	6232	91	14.60
11–20	3465	7	2.02
21–30	4338	18	4.15
31–40	4195	10	2.38
41–50	6224	21	3.37
51–60	8797	42	4.77
61–70	11354	60	5.28
71–80	11778	70	5.94
81-	4020	23	5.72
Gender			
Male	30964	182	5.88
Female	29439	160	5.43
Eye diseases^**∗**^			
Lens disorders	27602	160	5.80
Vitreous/retina diseases	9516	50	5.25
Ocular trauma	4479	16	3.57
Glaucoma	4300	28	6.51
Strabismus	3818	5	1.31
Ocular tumors	2366	47	19.86
Oculoplastics	2130	7	3.29
Cornea diseases	1971	16	8.12
Pterygium	1343	6	4.47
Lacrimal duct diseases	745	1	1.34
Orbital diseases	711	1	1.41
Uveitis	622	1	1.61
Others	800	4	5.00
*Total*	60403	342	5.66

^*∗*^ *P* < 0.001.

**Table 3:** Association of age and eye disease with the prevalence of critical results in eye patients by univariate logistic regression.

Characteristic	Category	OR (95% CI)	*P* value
Age (yrs)	0–10	2.46 (1.80; 3.36)	<0.001
	11–20	0.34 (0.16; 0.74)	0.007
	21–30	0.70 (0.42; 1.17)	0.16
	31–40	0.40 (0.21; 0.78)	0.007
	41–50	0.57 (0.35; 0.93)	0.02
	51–60	0.80 (0.55; 1.18)	0.26
	61–70	0.89 (0.63; 1.26)	0.51
	71–80	1 (reference)	
	81-	0.96 (0.60; 1.54)	0.88

Eye diseases	Lens disorders	1 (reference)	
	Vitreous/retina diseases	0.96 (0.70; 1.32)	0.80
	Ocular trauma	0.65 (0.39; 1.09)	0.10
	Glaucoma	1.19 (0.80; 1.78)	0.40
	Strabismus	0.24 (0.10; 0.58)	0.002
	Oncology	3.63 (2.62; 5.02)	<0.001
	Oculoplastics	0.60 (0.28; 1.28)	0.19
	Cornea diseases	1.48 (0.89; 2.48)	0.14
	Pterygium	0.82 (0.36; 1.84)	0.62

OR: odds ratio.

**Table 4:** Association of eye disease with the prevalence of critical results after adjusting for age and sex.

Eye disease	OR (95% CI)	*P* value
Lens disorders	1 (reference)	
Vitreous/retina diseases	0.79 (0.57; 1.10)	0.17
Ocular trauma	0.43 (0.25; 0.75)	0.003
Glaucoma	1.01 (0.66; 1.52)	0.95
Strabismus	0.14 (0.06; 0.35)	<0.001
Oncology	2.38 (1.64; 3.46)	<0.001
Oculoplastics	0.37 (0.17; 0.80)	0.01
Cornea diseases	1.14 (0.67; 1.93)	0.63
Pterygium	0.77 (0.34; 1.74)	0.53

OR: odds ratio.

## References

[1] Lundberg G. (1972). When to panic over an abnormal value. *Medical Laboratory Observer*.

[2] Doering T. A., Plapp F., Crawford J. M. (2014). Critical laboratory value thresholds. *American Journal of Clinical Pathology*.

[3] The Joint Commission. Accreditation Program (2012) Laboratory, 2012 National Patient Safety Goals (NPSG.02.03.01), http://www.jointcommission.org

[4] College of American Pathologists (2010) Laboratory General Checklist, http://www.cap.org

[5] Chinese Medical Doctor Association (2009). *Guide for Implementation of Patient Safety Goals (in Chinese)*.

[6] Clark E., Wald R., Levin A. (2012). Timing the initiation of renal replacement therapy for acute kidney injury in Canadian intensive care units: a multicentre observational study. *Canadian Journal of Anesthesia*.

[7] Joseph B., Aziz H., Zangbar B. (2014). Acquired coagulopathy of traumatic brain injury defined by routine laboratory tests: which laboratory values matter?. *Journal of Trauma and Acute Care Surgery*.

[8] Chen C.-I., Miser J., Kuan C.-F., Fang Y.-A., Lam C., Li Y.-C. (2013). Critical laboratory result reporting system in cancer patients. *Computer Methods and Programs in Biomedicine*.

[9] Schein O. D., Katz J., Bass E. B. (2000). The value of routine preoperative medical testing before cataract surgery. *The New England Journal of Medicine*.

[10] Cheng L., DeJesus A. Y., Rodriguez M. A. (2017). Using laboratory test results at hospital admission to predict short-term survival in critically Ill patients with metastatic or advanced cancer. *Journal of Pain and Symptom Management*.

[11] Dighe A. S., Rao A., Coakley A. B., Lewandrowski K. B. (2006). Analysis of laboratory critical value reporting at a large academic medical center. *American Journal of Clinical Pathology*.

[12] Yang D., Zhou Y., Yang C. (2013). Analysis of laboratory repeat critical values at a large tertiary teaching hospital in China. *PLoS ONE*.

[13] Piva E., Pelloso M., Penello L., Plebani M. (2014). Laboratory critical values: automated notification supports effective clinical decision making. *Clinical Biochemistry*.

[14] Grieme C. V., Voss D. R., Olson K. E., Davis S. R., Kulhavy J., Krasowski M. D. (2016). Prevalence and clinical utility of “incidental” critical values resulting from critical care laboratory testing. *Laboratory Medicine*.

[15] Chatterjee R., Zelnick L., Mukamal K. J. (2016). Potassium measures and their associations with glucose and diabetes risk: the multi-ethnic study of atherosclerosis (MESA). *PLoS ONE*.

[16] Harvey T. C. (2007). Addison's disease and the regulation of potassium: the role of insulin and aldosterone. *Medical Hypotheses*.

[17] Kocic I. (2012). Potassium channels as a target in smooth muscles and nerves. *Potassium Channels as a Target for Clinical Therapeutics*.

[18] Wolak T., Shoham-Vardi I., Sergienko R., Sheiner E. (2016). High potassium level during pregnancy is associated with future cardiovascular morbidity. *Journal of Maternal-Fetal and Neonatal Medicine*.

[19] He Q., Feng Y., Wang Y. (2015). Transient outward potassium channel: a heart failure mediator. *Heart Failure Reviews*.

[20] Tripodi A., Caldwell S. H., Hoffman M., Trotter J. F., Sanyal A. J. (2007). Review article: the prothrombin time test as a measure of bleeding risk and prognosis in liver disease. *Alimentary Pharmacology and Therapeutics*.

[21] Akdemir O. (2007). High blood glucose concerns heart specialist very.../coronary atherosclerosis distribution and the effect of blood glucose level on operative mortality/morbidity in diabetic patients undergoing coronary artery bypass grafting surgery: a single center experience. *Anadolu Kardiyoloji Dergisi*.

[22] Ambiru S., Kato A., Kimura F. (2008). Poor postoperative blood glucose control increases surgical site infections after surgery for hepato-biliary-pancreatic cancer: a prospective study in a high-volume institute in Japan. *Journal of Hospital Infection*.

[23] Chishaki A., Chishaki H. (2014). Postoperative high blood glucose - a potentially treatable marker related to atrial fibrillation after coronary artery bypass grafting. *Circulation Journal*.

[24] Liu R.-Y., Wang J.-J., Qiu X., Wu J.-M. (2014). Acute hyperglycemia together with hematoma of high-glucose blood exacerbates neurological injury in a rat model of intracerebral hemorrhage. *Neuroscience Bulletin*.

[25] Alcántara V., Cubero J. M., Prados A., Pérez Pérez J., Corcoy R. (2012). Seizure in a diabetic patient. Hypoglycemia or a side effect of continuous glucose monitoring?. *Endocrinología y Nutrición*.

[26] Terakawa Y., Tsuyuguchi N., Yamamura A., Nakagawa T. (2009). The influence of hypoglycemia on cerebral blood flow in cases of hypoglycemic coma. *Neurology India*.

[27] Chua W., Kho P. S., Moore M. M., Charles K. A., Clarke S. J. (2011). Clinical, laboratory and molecular factors predicting chemotherapy efficacy and toxicity in colorectal cancer. *Critical Reviews in Oncology/Hematology*.

[28] Eden P. R. (2015). Laboratory testing and pathological identification of cancer. *Medical Laboratory Observer*.

